# Enhanced Degradation of Paracetamol by the Fe(III)-Sulfite System under UVA Irradiation

**DOI:** 10.3390/molecules27072248

**Published:** 2022-03-30

**Authors:** Yanan Yuan, Feng Wu, Marcello Brigante, Gilles Mailhot

**Affiliations:** 1 Yichang Atmospheric Pollution Prevention and Control Administrative Center, Yichang 435000, China; yuanyn0829@sina.com; 2 Department of Environmental Science, School of Resources and Environmental Science, Wuhan University, Wuhan 430079, China; 3 Institut de Chimie de Clermont-Ferrand, Université Clermont Auvergne, CNRS, Clermont Auvergne INP, F-63000 Clermont-Ferrand, France; marcello.brigante@uca.fr

**Keywords:** paracetamol, photodegradation, Fe(III), sulfite ions, sulfate radical

## Abstract

The Fe(III)-S(IV) system used for advanced oxidation processes (AOPs) at acidic pH has just been proposed and demonstrated valid for very few contaminants in the last several years. In this work, we investigated the effect of ultraviolet A (UVA) radiation on the degradation efficiency of the Fe(III)/S(IV) system at near-neutral pH. Paracetamol (PARA) was selected as a model contaminant. The influencing factors, such as initial pH and Fe(III)/S(IV) molar ratio on chemical kinetics, and the mechanism of PARA degradation are investigated, with an emphasis on the determination of dominant oxidant species. Our results show that irradiation enhances the PARA degradation by accelerating the decrease of pH to acidic levels, and the optimal pH for the degradation of PARA in the Fe(III)/S(IV)/O_2_ system was around 4.0. At near-neutral pH, more than 60% of PARA was decomposed within 40 min under irradiation, whereas no significant degradation of PARA was observed using Fe(III)/S(IV) at pH 7.0 without irradiation. Mechanism investigation revealed that sulfate radical (SO_4_^•‒^) is the main oxidant species generated and responsible for the PARA degradation under these conditions. This finding may have promising implications in developing a new degradation process for dealing with wastewater at near-neutral pH by the Fe(III)/S(IV)/O_2_ system under UVA irradiation.

## 1. Introduction

The presence of pharmaceuticals and personal care products (PPCPs) in water compartments becomes an emerging problem for our society with a recognized risk for the environment and human health [1,2]. Numerous studies reported that conventional wastewater treatments are not able to provide an efficient or complete degradation of such active molecules. In the last two decades, different physico-chemical processes able to provide an efficient abatement of active organic molecules in water were investigated. Among them, advanced oxidation processes (AOPs) are a class of new and promising decontamination approaches with, as a common point, the formation of strong oxidant species, such as radicals. Considering the different class of AOPs, sulfur radicals based AOPs (SR-AOPs) have attracted widespread attention due to high selectivity and strong oxidative potential of sulfate radical toward organic pollutants [3]. Sulfate radical (SO_4_^•‒^, E0 = 2.5–3.1 V vs. NHE) is generated using thermal, photochemical, and electrochemical activation of precursors, such as persulfate (S_2_O_8_^2−^, PS), peroxymonosulfate (HSO_5_^−^, PMS), and sulfite (SO_3_^2−^) ions [4]. Recently, the use of transition metal ions (TMI) in SR-AOPs has been reported to be a catalytic process, leading to the efficient abatement of organic pollutants in water [5]. In a recent work, we reported the rapid degradation of aniline when ferric ions (Fe^3+^) was used in the presence of SO_3_^2−^ in an optimum initial pH of 4.0 [6]. However, the degradation efficiency was less than 10% at an initial pH of 7.0 and a plateau was reached after ~20 min of reaction. The mechanism of the Fe(III)/S(IV) system and involvement of reactive species follow different activation steps in which formation of sulfite/iron complexes (R1–4) leads to the formation of sulfur reactive species (SRS) (R5–10).

Fe^3+^ + HSO_3_^−^ ↔ FeSO_3_^+^ + H^+^ (R1)

FeSO_3_^+^ ↔ Fe^2+^ + SO_3_^•‒^ (R2)

Fe^2+^ + HSO_3_^−^ → FeHSO_3_^+^ (R3)

4FeHSO_3_^+^ + O_2_ → 4FeSO_3_^+^ + 2H_2_O (R4)

SO_3_^•‒^ + O_2_ → SO_5_^•‒^ (R5)

SO_5_^•‒^ + HSO_3_^−^ → SO_3_^•‒^ + HSO_5_^−^ (R6)

SO_5_^•‒^ + HSO_3_^−^ → SO_4_^•‒^ + H^+^ + SO_4_^2−^ (R7)

SO_4_^•‒^ + HSO_3_^−^ → SO_3_^•‒^ + H^+^ + SO_4_^2−^ (R8)

SO_5_^•‒^ + SO_5_^•‒^ → 2SO_4_^•‒^ + O_2_ (R9)

Fe^2+^ + SO_5_^•‒^ + H^+^ → Fe^3+^ + HSO_5_^−^ (R10)

In order to improve the degradation efficiency of the Fe(III)/S(IV) system, in this work, we explored the effect of UVA radiation and radical formation enhancement at near-neutral pH. The use of UVA that is a fraction of sun radiation demonstrates the possible use of solar light for application in a real scale remediation process. For this purpose, paracetamol (PARA), a widely used analgesic and antipyretic drug and an important material for the manufacturing of azo dyes, was selected as a model pollutant due to its presence in natural water and wastewater [7]. Reactions at pH 4.0 and 7.0, with or without UVA irradiation, have been compared to provide mechanistic insights. The effects of initial pH, Fe(III)/S(IV) molar ratio, oxygen content, and buffer have been investigated to determine the optimum conditions for this novel photochemical process. Moreover, radical scavenger experiments have been conducted to determine the dominant oxidant species in such systems.

## 2. Results

### 2.1. Control Experiments

**Figure 1:**
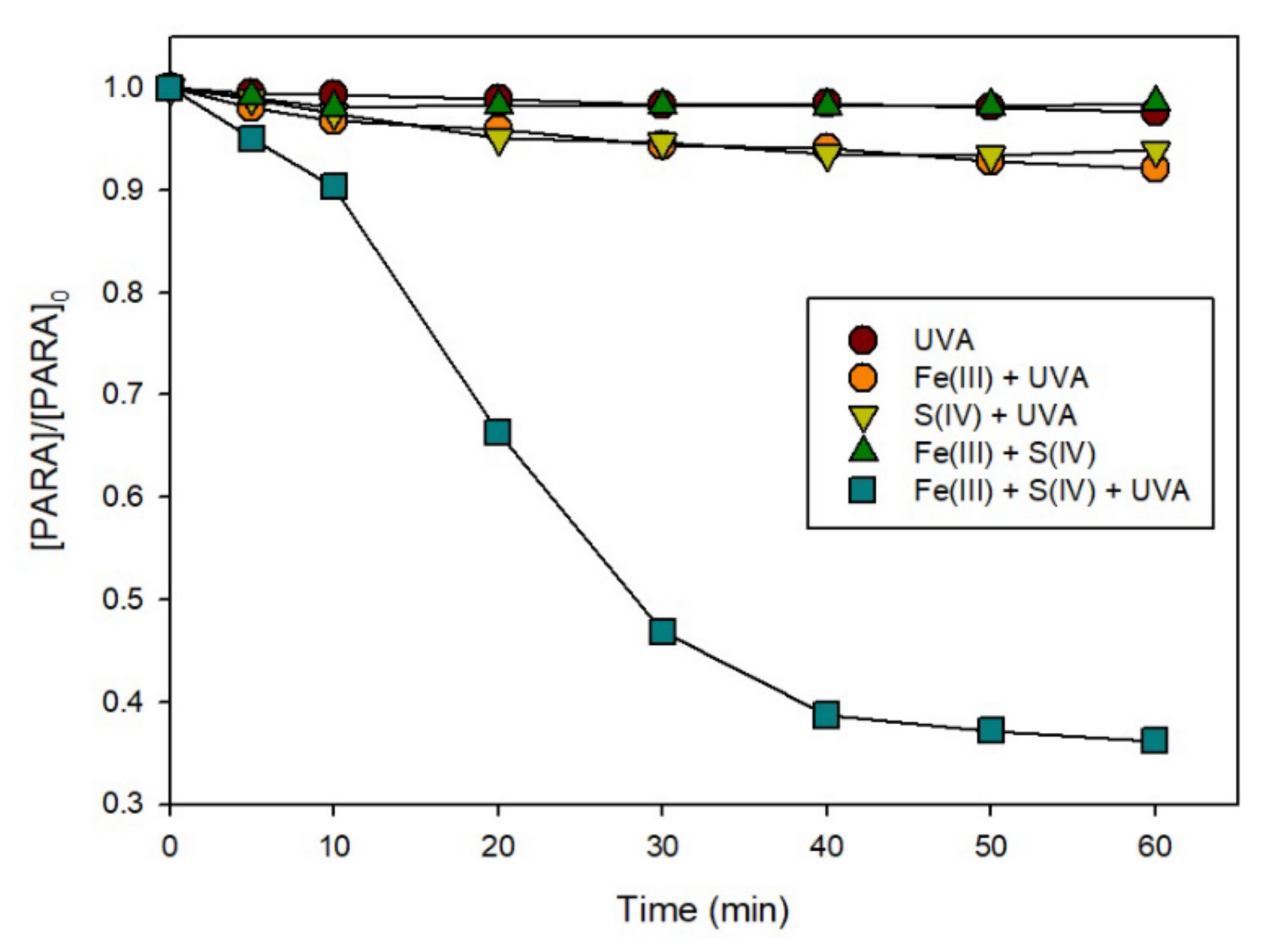
Degradation of PARA under various conditions in aerated solutions. Conditions: [PARA]_0_ = 10 μM, [Fe(III)]_0_ = 0.1 mM, [S(IV)]_0_ = 1.0 mM, pH_init_ = 7.0.

To evaluate the performance of the Fe(III)/S(IV)/O_2_/UVA system, the degradation of PARA in an aqueous solution at near-neutral pH was investigated. Figure 1 shows a comparison of the extents of PARA degradation under the different conditions. UVA, UVA/S(IV), UVA/Fe(III), and Fe(III) + S(IV) at pH 7.0 did not lead to significant degradation of PARA (less than 10% after 60 min). As no photochemical degradation of PARA was observed in the absence of sulfite ions, it can be inferred that the reaction between Fe(III) ions and oxygen under irradiation to produce reactive oxygen species does not contribute to the degradation of PARA at near-neutral pH. Hence, the enhancement by irradiation has no effect on the photochemical reaction of free Fe(III) ions at near-neutral pH, while only UVA/Fe(III)/S(IV) at pH 7.0 leads to the degradation of PARA. When the reaction was conducted under irradiation with 0.1 mM Fe(III) and 1.0 mM S(IV) in the solution (Fe(III)/S(IV)/O_2_/UVA), more than 60% of PARA were decomposed within 40 min.

**Figure 2:**
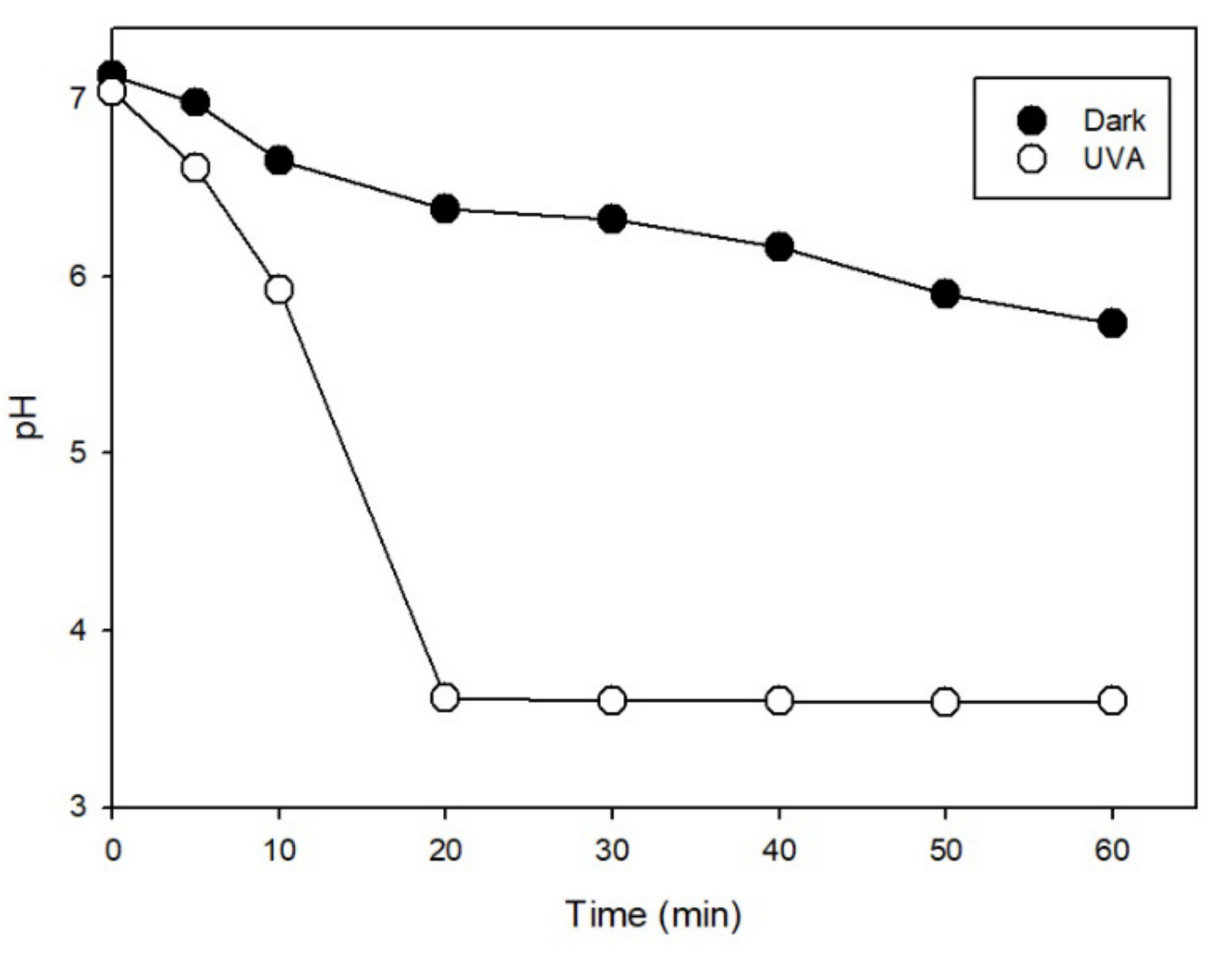
Change of pH during reaction in aerated solutions. Conditions: [PARA]_0_ = 10 μM, [Fe(III)]_0_ = 0.1 mM, [S(IV)]_0_ = 1.0 mM, pH_init_ = 7.0.

The degradation mechanism was initiated by the Fe(III)/S(IV) complex formation (FeSO_3_^+^) [8,9,10,11], which then underwent ligand-to-metal charge transfer (LMCT) reaction [12] to generate SO_3_^•−^ [13]. On the other hand, monitoring of the pH during the reaction revealed a much greater decrease under irradiation, as shown in Figure 2. The pH decreased slightly during the initial stage of the reaction and then decreased sharply between 10 and20 min. Moreover, the rate of PARA degradation (Figure 1) varied in the same way as the decrease in pH. The results indicate that irradiation can enhance the reaction in the Fe(III)/S(IV)/O_2_/UVA system, lowering the pH from near-neutral to moderately acidic (pH 3.5).

### 2.2. Effect of pH on the Degradation of PARA

**Figure 3:**
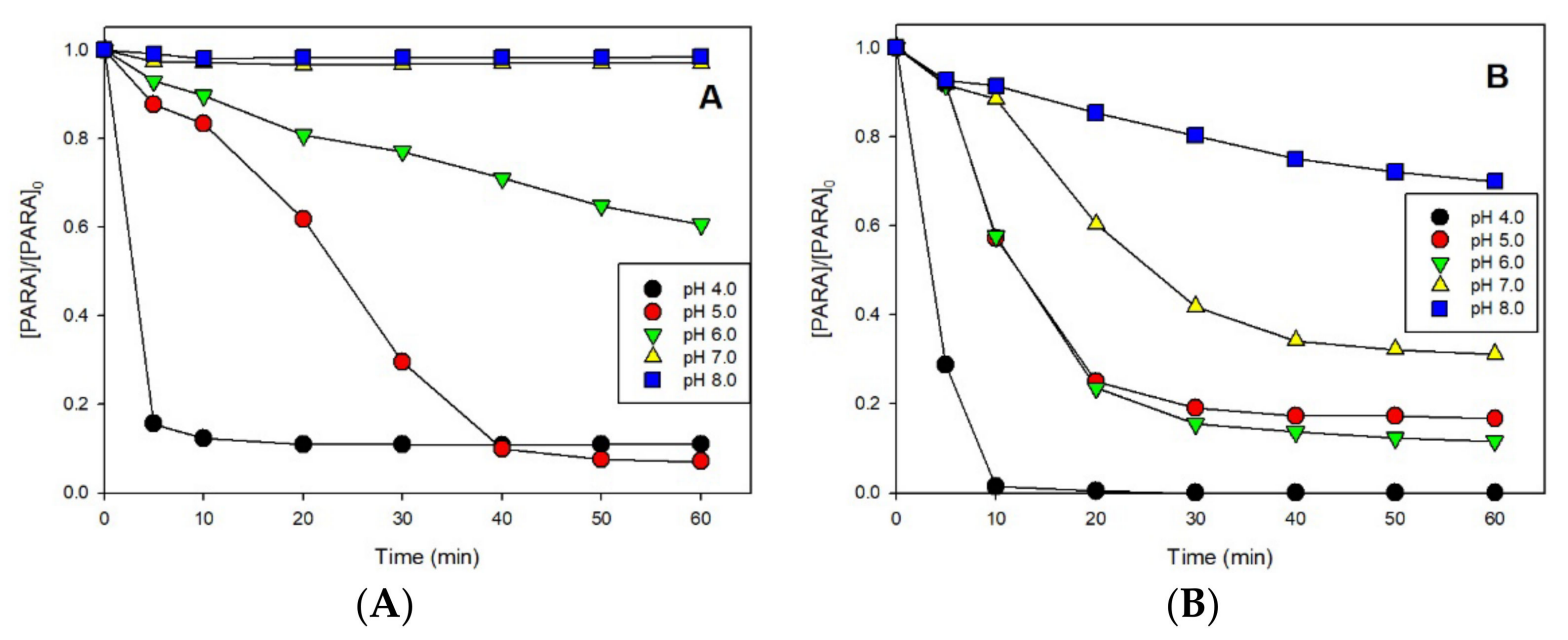
Effect of pH on PARA degradation under dark (**A**) and UVA (**B**) in aerated solutions. Conditions: [PARA]_0_ = 10 μM, [Fe(III)]_0_ = 0.1 mM, [S(IV)]_0_ = 1.0 mM.

To determine the effect of pH on the degradation of PARA, samples were analyzed at initial pH values ranging from 4.0 to 8.0, under dark and irradiation. Figure 3A shows that, without irradiation, the efficiency of PARA degradation decreased with increasing initial pH. With an initial pH 6.0, the degradation efficiency of PARA was only 39% after 60 min of reaction. When the initial pH of the solution was ≥7.0, there was no PARA degradation. Corresponding experiments were performed under UVA irradiation in the same pH range of 4.0 to 8.0. As shown in Figure 3B, the efficiency of PARA degradation also decreased with increasing pH. However, the reaction proceeded at pH 7.0, and after 60 min, the degradation efficiency reached around 69%. At pH ≥ 8.0, PARA degradation was still evident, with more than 25% of disappearance after 60 min of irradiation. Comparing the efficiencies of PARA degradation with or without irradiation, the reaction rate under irradiation was very fast and complete degradation was achieved at pH 4.0, whereas it proceeded to a lower extent in the dark. This result is consistent with earlier reports by our group of greatly enhanced rates of contaminant degradation with the Fe(III)/S(IV) system [9,14,15] under irradiation. In the range of pH tested, the optimum initial pH of the Fe(III)/S(IV) system is 4.0. When the initial pH of the solution was increased to 5.0, the initial reaction rate of PARA degradation under irradiation was much faster than that without irradiation, but after 60 min, the degradation efficiencies were comparable. This was due to the decrease in pH, which enhanced the reaction. However, at pH > 6.0, the difference between PARA degradation with or without irradiation became much more marked. For example, at pH 6.0, the degradation efficiencies were around 39% and 83% without and with irradiation, respectively. This result corroborates that irradiation greatly enhances the reaction, which can be explained by the associated decrease in pH to acidic values. This phenomenon implies that photodegradation of organic compounds should be considered as an important pathway in media at near-neutral pH.

When the solution was buffered at pH 7.0 by adding PIPES, the results shown in Supplementary Materials: Figure S1 indicate that almost no PARA degradation was achieved. This result is in agreement with the above-mentioned pH effect. Efficiency of the reaction strongly depends on the decrease in pH induced by irradiation. In the presence of a buffer, the system cannot attain the appropriate pH level to facilitate the reaction.

### 2.3. Effect of Fe(III)/S(IV) Concentrations on the Degradation of PARA

**Figure 4:**
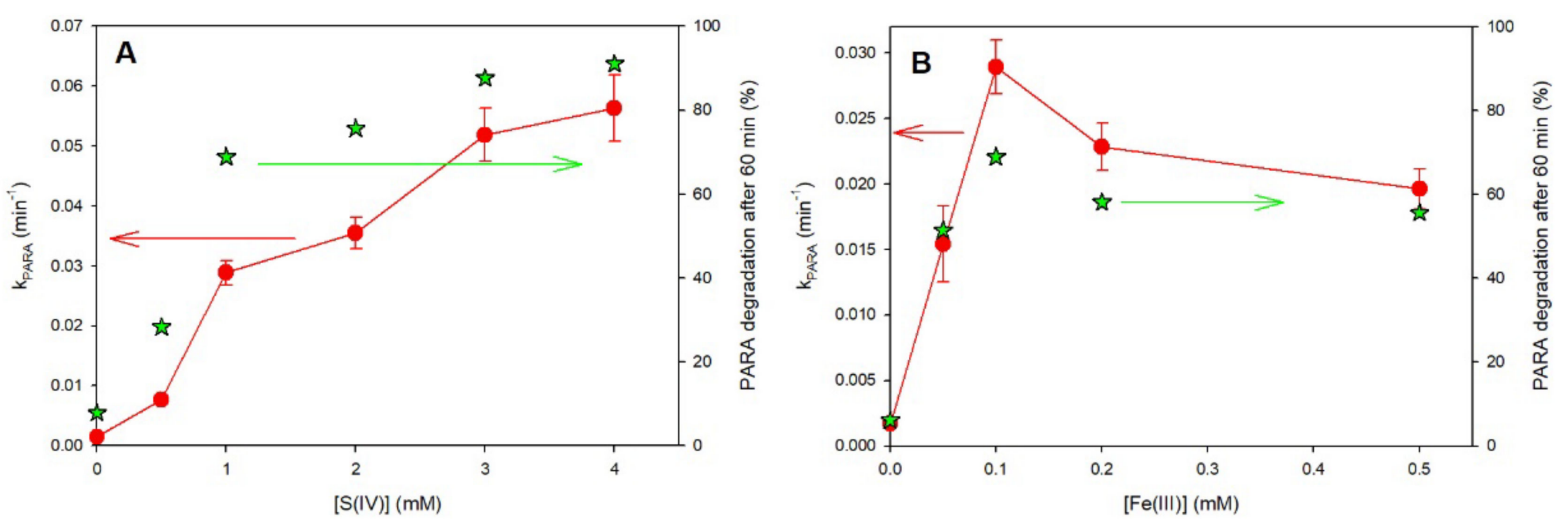
First order rate constant of PARA degradation (k_PARA_) (filled circles) and degradation efficiency in percentage after 60 min (green stars) as a function of S(IV) (**A**) and Fe(III) (**B**) concentrations under UVA in aerated solutions. Conditions: [PARA]_0_ = 10 μM, [Fe(III)]_0_ = 0.1 mM, [S(IV)]_0_ = 1.0 mM, pH_init_ = 7.0.

The efficiencies of PARA degradation at various Fe(III) or S(IV) concentrations, at an initial PARA concentration of 10 µM and an initial pH of 7.0, were determined. The results in Figures S2 and S3 clearly show that the Fe(III) and S(IV) concentrations greatly influenced the efficiency of PARA degradation. The first order rate constant of PARA degradation (k_PARA_) increased from 1.44 ± 0.23 × 10^−3^ min^−1^ when no S(IV) was present in the solution up to 5.63 ± 0.56 × 10^−2^ min^−1^ using 4 mM of S(IV) for an initial Fe(III) concentration at 0.1 mM (Figure 4A), corresponding to a PARA degradation efficiency after 60 min of irradiation of 7.9% to 91.0%, respectively. However, previously reported works [9,16] indicated that, at higher sulfite ion concentrations, no significant enhancement of the pollutant degradation can be observed due to the reactivity competition of SO_4_^•−^/SO_5_^•−^ between the contaminant and SO_3_^2−^ (R7 and 8). In the present case, we observed no inhibition with excess of sulfite ions, indicating that photochemical reactions involving the consumption of sulfite became more significant than inhibition reactions by excess sulfite. Considering its potential for causing serious pollution, application, and feasibility of use in industrial processes or engineered systems, an S(IV) dosage of 1.0 mM was used when other individual effects were investigated.

Batch experiments under irradiation with a fixed initial concentration of S(IV) (1.0 mM) were conducted to investigate the effects of initial concentrations of Fe(III) in the range from 0 to 0.5 mM. As can be seen from Figure 4B, an increase in Fe(III) concentration up to 0.1 mM improved the degradation of PARA up to 69% after 60 min. On the contrary, when the Fe(III) concentration exceeded 0.1 mM, an inhibition can be observed. This negative effect can be attributed to the large amount of Fe(II) generated in the iron cycle and its reaction with sulfate radicals [17] and SO_5_^•−^ (R10). These results show that, to efficiently remove PARA from aqueous solution, a small amount of Fe(III) is quite sufficient to obtain good degradation efficiency.

### 2.4. Effects of Oxygen

**Figure 5:**
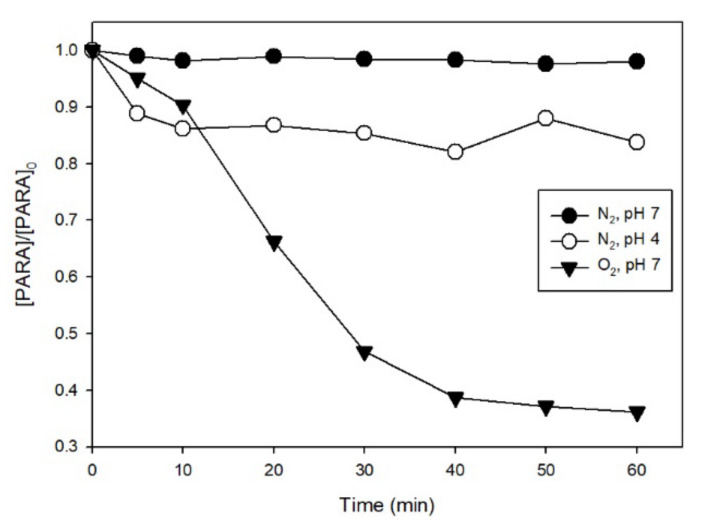
Effect of oxygen on PARA degradation with UVA irradiation. Conditions: [PARA]_0_ = 10 μM, [Fe(III)]_0_ = 0.1 mM, [S(IV)]_0_ = 1.0 mM (the PARA degradation at pH 4.0 with O_2_ is depicted in Figure 3B).

The effect of oxygen on the degradation of 10 µM PARA was studied using 0.1 mM Fe(III) and 1.0 mM S(IV) at both pH 4.0 and 7.0 under UVA irradiation. As shown in Figure 5, in de-aerated solutions, there was almost no degradation at pH 7.0 and only a 16% loss of PARA at pH 4.0 after 60 min of irradiation. This percentage of PARA degradation is much smaller than the 64% loss at pH 7.0 and complete degradation at pH 4.0 obtained in aerated solutions (Figure 3B). The results show oxygen level to be a very important parameter affecting the photodegradation of PARA in the Fe(III)/S(IV)/O_2_/UVA system at different pH. Thus, we surmise that oxygen takes part in the photochemical process in this system. The role of oxygen is to generate SO_4_^•−^ and SO_5_^•−^ in solution by reaction with SO_3_^•−^ (R5–9), and the observation that there is still some degradation of PARA at pH 4.0 without oxygen can be ascribed to the generation of some hydroxyl radical (HO^•^) at pH 4.0 under irradiation (R11) [18,19,20].

### 2.5. Effect of Radical Scavengers

To further evaluate the reaction mechanism in the studied system, radical scavenger experiments were conducted using EtOH and TBA to identify the primary generated radicals at pH 4.0 and 7.0. Reactivity constants of HO^●^ and SO_4_^●−^ with both quenchers and PARA were considered in order to estimate the quenching ratio and then the contribution of each radical to the PARA degradation (see Table 1).

Using 0.1 M of EtOH in the presence of 10 µM of PARA, we can estimate that more than 99.9% of HO^●^ and 97 % of SO_4_^•−^ are quenched in solution, while the use of 2 mM of TBA allows a more selective quenching of HO^●^ (90%) compared to the SO_4_^●−^ (~2.5%).

**Figure 6:**
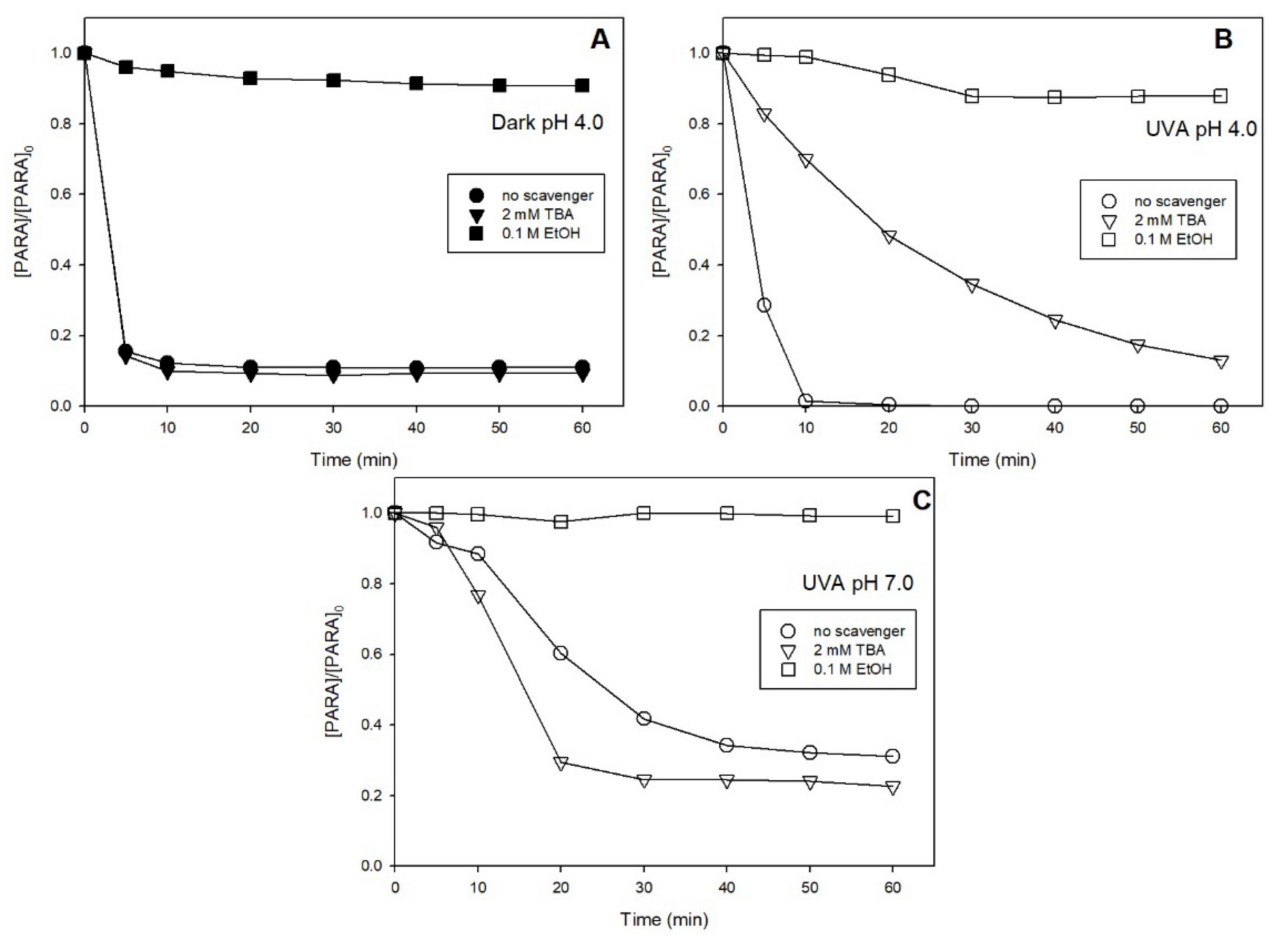
Effect of radical scavengers on PARA degradation in the dark at pH 4.0 (**A**) and under UVA at pH 4.0 (**B**) and 7.0 (**C**). Conditions: [PARA]_0_ = 10 μM, [S(IV)]_0_ = 1.0 mm, [Fe(III)]_0_ = 0.1 mM.

At pH 4.0, under dark condition in Figure 6A, almost no effect of TBA was observed, while complete PARA degradation inhibition occurred in the presence of EtOH, indicating that mainly SO_4_^●−^ was generated in this system. However, under UVA (Figure 6B), the degradation of PARA was partially inhibited, also in the presence of TBA, suggesting the generation of both radicals HO^●^ and SO_4_^●−^.

At pH 4.0, the generation of HO^●^ cannot be attributed to the reaction between SO_4_^●−^ and H_2_O, which has a negligible reactivity constant, while the involvement of FeOH^2+^ photolysis in an acidic environment can be considered as the main source (R11) [10]. SO_4_^●−^ derives from the decomposition of FeSO_3_^+^ complexes and the oxidation of SO_3_^●‒^ (R12).

FeOH^2+^ + UV → Fe^2+^ + HO^●^ (R11)

FeSO_3_^+^ + UV → Fe^2+^ + SO_3_^●−^ (R12)

Figure 6C shows the effect of TBA and EtOH to the Fe(III)/S(IV)/O_2_/UVA system at pH 7.0. Interestingly, a PARA degradation increase was observed when 2 mM of TBA were added to the solution. Such an effect should be explained considering that HO^●^ is also involved in the redox cycle of Fe^2+^/Fe^3+^ in the solution, improving the formation of Fe^3+^ and thus decreasing the concentration of Fe^2+^ in the solution. The decrease of Fe^2+^ concentration limits its reactivity with SO_5_^•−^ (R10) and therefore can explain the higher degradation of PARA. On the contrary, in the presence of 0.1 M of EtOH, almost no PARA degradation occurs, suggesting that SO_4_^•−^ is really the main radical generated at pH 7.0.

**Figure 7:**
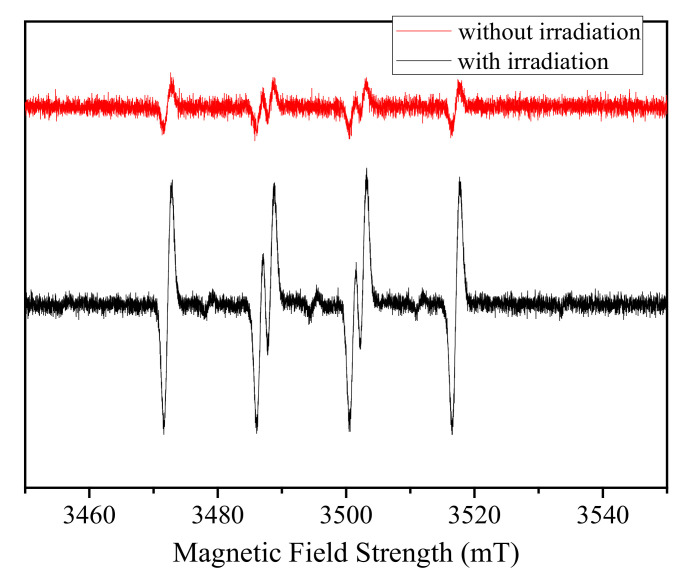
ESR spectra of reaction solutions at pH 7.0. Conditions: [S(IV)]_0_ = 1.0 mM, [Fe(III)]_0_ = 0.1 mM. [DMPO] = 1 g L^−1^.

To gain further evidence for the mechanism in the Fe(III)/S(IV)/O_2_/UVA system, ESR spectra were recorded to identify radical intermediates formed during reactions of Fe(III)/S(IV) using DMPO as a spin trap. SO_4_^•−^ and HO^•^ were detected by measuring the signals of their respective DMPO−HO^•^ and DMPO−SO_4_^•−^ adducts, as described previously [24,25]. As shown in Figure 7, for the Fe(III)/S(IV)/UVA system, the hyperfine coupling constants in the measured spectra were consistent with those of DMPO–SO_3_^•−^ adducts, and no DMPO−OH adducts were detected. These observations confirm that the HO^•^ concentration is insignificant under these conditions and that SOx^•−^ (including SO_3_^•−^, SO_4_^•−^, and SO_5_^•−^) are the primary reactive oxidants responsible for PARA degradation. However, in the absence of DMPO and in the presence of oxygen, SO_3_^•−^ can be easily oxidized to SO_5_^•−^ (R5), thereby considerably mitigating the contribution of SO_3_^•−^ in our system. Combining the results of the radical scavenger experiments, the fact that EtOH can completely inhibit the reaction and its negligible reactivity with SO_5_^•−^ (<1 × 10^3^ M^−1^ s^−1^) [26], it can be concluded that, in the Fe(III)S(IV)/O_2_/UVA system, SO_4_^•−^ is the oxidant that is mainly responsible for the degradation of PARA.

**Scheme 1:**
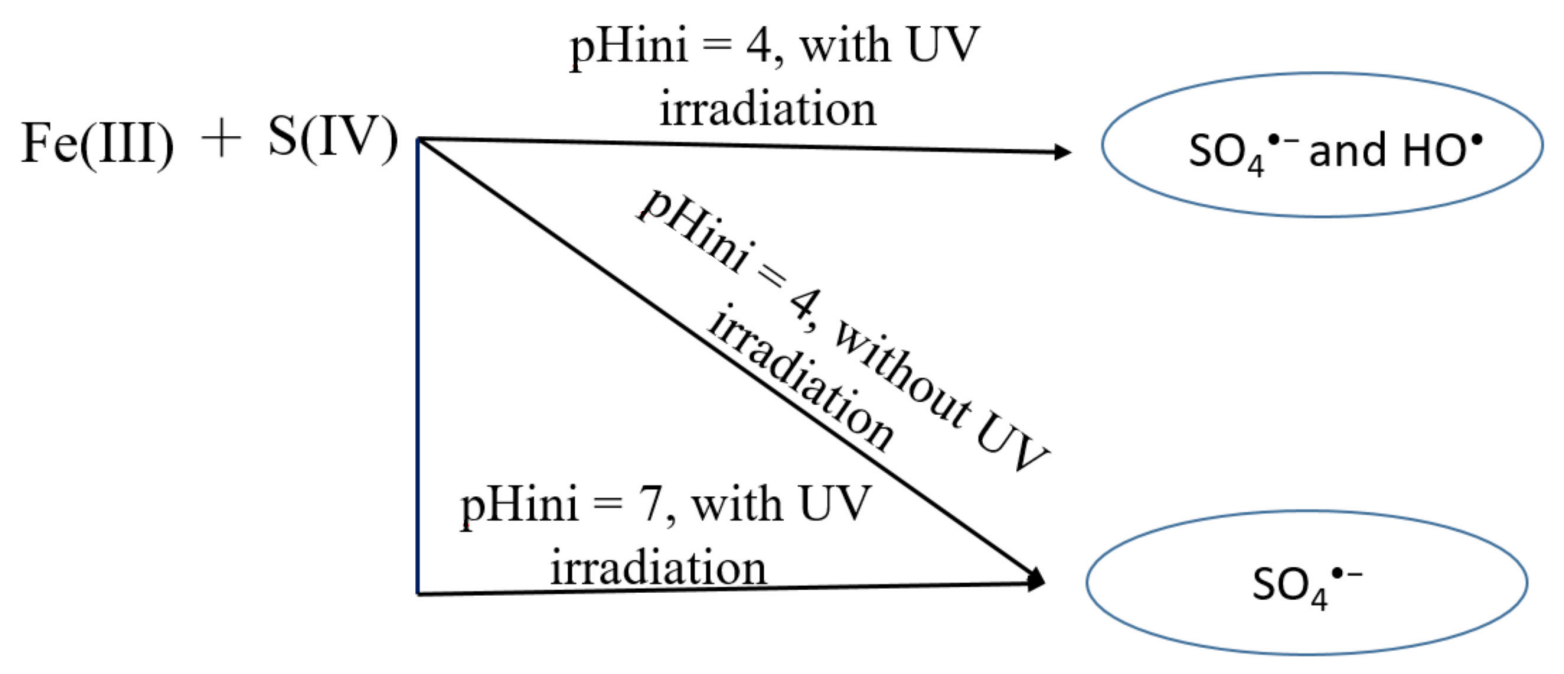
Proposed mechanism in the Fe(III)/S(IV) system at pH 4.0 and 7.0 under dark and UVA irradiation.

Scheme 1 shows a schematic diagram of the proposed pathways in the Fe(III)/S(IV)/O_2_/UVA system at pH 7.0 compared with the Fe(III)/S(IV)/O_2_ system at pH 4.0, with or without UVA irradiation, for the degradation of PARA. SO_4_^•−^ and HO^•^ radicals are generated under UVA irradiation at initial pH 4.0. The generation of HO^•^ is mainly due to the photolysis of iron aquacomplexes, as described in numerous studies [27]. SO_4_^•−^ is also generated at pH 4.0 without irradiation. Its presence is due to the formation of sulfite/iron complexes and their equilibria with sulfur reactive species (sulfur radicals). However, at pH 7.0, no iron aquacomplexes are present (i.e., formation of soluble iron aggregates and/or iron hydroxides and consequent precipitation occurs under neutral pH values) and only SO_4_^•−^ are detected under UVA irradiation.

## 3. Materials and Methods

### 3.1. Chemicals

Iron(III) perchlorate hydrate (Fe(ClO_4_)_3_·xH_2_O), paracetamol (PARA), 5,5-dimethyl-1-pyrroline N-oxide (DMPO), and sodium sulfite (Na_2_SO_3_) were obtained from Sigma, France, like all chemicals used in this work, unless otherwise stated. Tert-Butyl alcohol (TBA) and ethanol (EtOH) were used as scavengers to quench the relevant radicals. Piperazine-N,N′-bis (2-ethanesulfonic acid) (PIPES) was used as a buffer to keep solutions at pH 6.7–7.0. NaOH and HClO_4_, were used to adjust the pH of solutions. All the pHs mentioned in the work are initial pH and can evolve during the experiment, except where the use of a buffer is specified to keep the pH constant. Ferrozine (3-(2-pyridyl)-5,6-diphenyl-1,2,4-triazine-p,p′-disulfonic acid monosodium salt hydrate) and ascorbic acid, were used to determine the concentrations of Fe(II) and Fe(III). Methanol (HPLC grade) was purchased from Fluka. All chemicals were used without further purification.

### 3.2. Experimental Setup

Photochemical experiments were carried out in a Pyrex photoreactor placed in a cylindrical stainless-steel container equipped with four fluorescent tubes (Philips TL D 15W/05 with a continuous emission in the 300–500 nm). The actual light intensity emission between 300–370 nm (Io_300–370 nm_) was determined as around 540 W m^−2^ by chemical actinometry [28]. The temperature was kept constant at 293 K by circulating thermostatically controlled water through an outer jacket. Predetermined amounts of PARA and Fe(III) stock solutions were diluted in a 100 mL flask and transferred to the reactor after complete mixing. The pH was adjusted to the desired value by adding concentrated solutions of NaOH or HClO_4_. Na_2_SO_3_ solution (the pH of which was pre-adjusted according to the reaction) was then added to the reaction solution to reach the desired concentration, and then the lamp was switched on. At specified time intervals, 1 mL of solution was withdrawn from the reactor for analysis. Pure O_2_ (99.99%) was continuously bubbled through the solutions to maintain oxygen saturation. In N_2_ purging experiments, solutions were purged with high-purity N_2_ (99.99%) by bubbling for 30 min prior to reaction, and the bubbling was maintained during the reaction. In the radical scavenging experiments, specific concentrations of TBA or EtOH, scavengers for HO^•^ or SO_4_^•−^, respectively, were added to the reaction solutions. In the buffer experiment, predetermined amounts of PIPES reagent were added to the solution at the same time with substrate, and the remaining procedure was analogous to that described above.

### 3.3. Analytical Methods

The concentration of PARA was determined using a Shimadzu LC-10A high-performance liquid chromatography (HPLC) system equipped with a UV/Vis detector (SPD-10AV; Shimadzu) and an ODS-C18 column (25 cm × 4.6 mm, 5 μm; Shimadzu, Kyoto, Japan). The mobile phase was a mixture of methanol/water (25:75, *v*/*v*) at a flow rate of 1.0 mL min^−1^. The detector was set at 241 nm, corresponding to the maximum absorption of PARA. Concentration of PARA during degradation was fitted by the following first order equation: C_t_/C_0_ = exp (−k’t) where C_0_ and C_t_ are, respectively, the initial concentration and the concentration at time t and k’ is the pseudo-first order rate constant. The error associated with the kinetic constant data represent 3σ, derived from the scattering of the experimental data around the fit curves (intra-series variability).

Fe(II) and Fe(III) concentrations were determined by complexation with Ferrozine [7]. Firstly, Fe(II) concentration was determined by measuring the absorption at 562 nm of a solution obtained by mixing sample (1 mL), water (4 mL), and 20 mM Ferrozine solution (500 μL). Considering that the molar absorption coefficient of the complex Fe(II)-Ferrozine at 562 nm is equal to M^−1^ cm^−1^, the concentration of Fe(II) can be calculated according to Equation (1):

(1) $$\mathrm{Fe}\left(\mathrm{II}\right)=\frac{A_{sample}-A_{blank}}{\varepsilon_{562}\times l}\times \frac{V_{tot}}{V_{sample}}$$

To determine the total Fe concentration (Fe(II) + Fe(III)), it was necessary to reduce Fe(III) to Fe(II), for which a 0.5 M stock solution of ascorbic acid was used. Sample (1 mL) and water (4 mL) were mixed with 600 μL of this ascorbic acid solution, and after 20 min, 500 µL of Ferrozine solution were added.

## 4. Conclusions

The photodegradation of PARA has been investigated in the Fe(III)/S(IV)/O_2_ system in the absence and presence of UVA irradiation. The results indicate that pH, Fe(III)/S(IV) concentration, and oxygen content all have important impacts on the degradation of PARA. To achieve a higher efficiency of PARA degradation, a higher S(IV) concentration is favorable. Irradiation enhances the degradation of PARA by accelerating the decrease of pH to acidic levels, and the optimal pH for the degradation of PARA in the Fe(III)/S(IV)/O_2_ system is about 4.0. Adding TBA to the suspension has almost no inhibitory effect on PARA degradation, but when EtOH is added, the degradation of PARA is almost completely inhibited. These results, combined with those of ESR experiments, indicate that the primary degradation pathway of PARA is due to its reaction with sulfate radical, SO_4_^•−^. In conclusion, UVA irradiation can be used to enhance the degradation of PARA by the Fe(III)/S(IV)/O_2_ system with initial pH around 7.0. These attributes make the Fe(III)/S(IV)/O_2_/UVA system more promising to deal with wastewater at near-neutral pH.

## Figures and Tables

**Table 1:** The second order rate constants of HO^●^ and SO_4_^●−^ with both quenchers (EtOH and TBA) and PARA [21,22,23].

	PARA	EtOH	TBA
$k_{HO^{•}}$ (M^−1^ s^−1^)	1.35 × 10^10^	1.9 × 10^9^	6.0 × 10^8^
$k_{SO_{4}^{•-}}$ (M^−1^ s^−1^)	6.44 × 10^9^	4.3 × 10^7^	8.4 × 10^5^

## Data Availability

Not applicable.

## Supplementary Materials

The following supporting information can be downloaded at: https://www.mdpi.com/article/10.3390/molecules27072248/s1, Figure S1: Effect of buffer on PARA degradation with UVA irradiation in aerated solutions. Conditions: [PARA]_0_ = 10 μM, [S(IV)]_0_ = 1.0 mM, [Fe(III)]_0_ = 0.1 mM, pHinit = 7.0. Figure S2: Effect of S(IV) concentration on PARA degradation with UVA irradiation in aerated solutions. Conditions: [PARA]_0_ = 10 µM, [Fe(III)]_0_ = 0.1 mM, pHinit = 7.0. Figure S3: Effect of Fe(III) concentration on PARA degradation with UVA irradiation in aerated solutions. Conditions: [PARA]_0_ = 10 µM, [S(IV)]_0_ = 1.0 mM, pHinit = 7.0.

## Sample Availability

Samples of the compounds are not available from the authors.
