# Supplementary material for: Enhanced Degradation of Paracetamol by the Fe(III)-Sulfite System under UVA Irradiation
Source: Molecules. 2022 Mar 30;27(7):2248. doi: 10.3390/molecules27072248 (PMC9000444; doi:10.3390/molecules27072248)
Supplement: Supplementary file 1 [file molecules-27-02248-s001.zip › molecules-1608248-supplementary.pdf]

SUPPLEMENTARY MATERIAL

Enhanced degradation of paracetamol by the Fe(III)-sulfite system under UVA irradiation

Yanan Yuan[1], Feng Wu[2*], Marcello Brigante[3], Gilles. Mailhot[3*]

[1] Yichang Atmospheric Pollution Prevention and Control Administrative Center, Yichang, 435000, China.

[2] Department of Environmental Science, School of Resources and Environmental Science, Wuhan University, Wuhan, 430079, China.

[3] Université Clermont Auvergne, CNRS, Clermont Auvergne INP, Institut de Chimie de Clermont-Ferrand, F-63000 Clermont-Ferrand, France.

Corresponding Authors: Feng Wu (fengwu@whu.edu.cn) and Gilles Mailhot (gilles.mailhot@uca.fr)

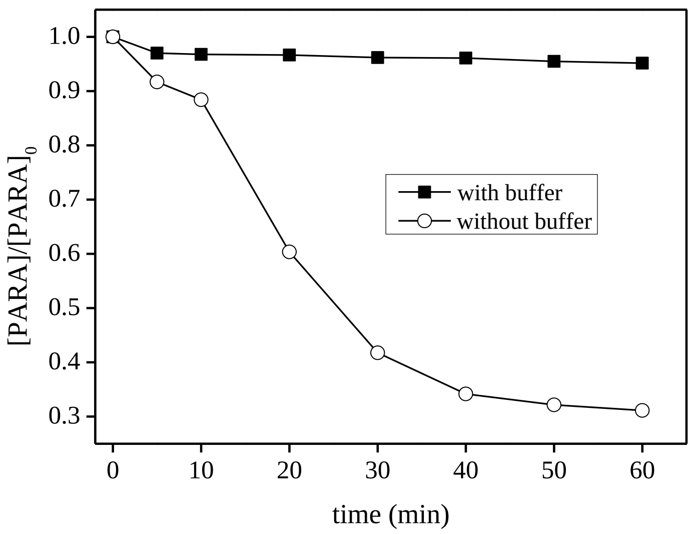

Figure S1. Effect of buffer on PARA degradation with UV irradiation in aerated solutions. Conditions: $[PARA]_0$ = 10 μM, $[S(IV)]_0$ = 1.0 mM, $[Fe(III)]_0$ = 0.1 mM, pH_{init} = 7.0.

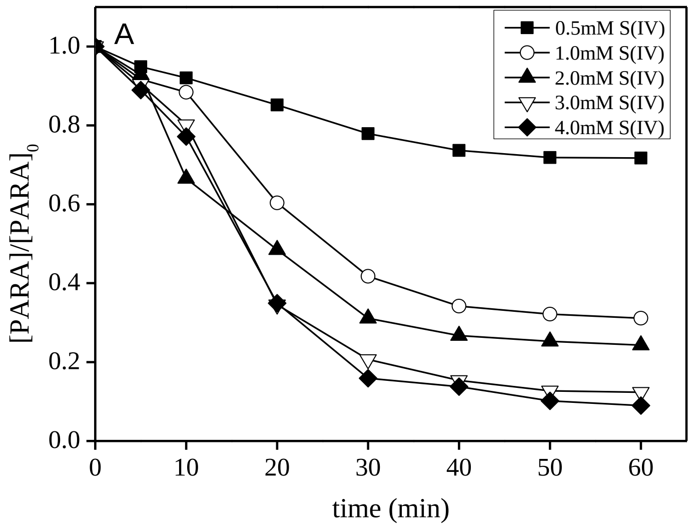

Figure S2. Effect of S(IV) concentration on PARA degradation with UV irradiation in aerated solutions. Conditions: $[PARA]_0$ = 10 µM, $[Fe(III)]_0$ = 0.1 mM, pH_{init} = 7.0.

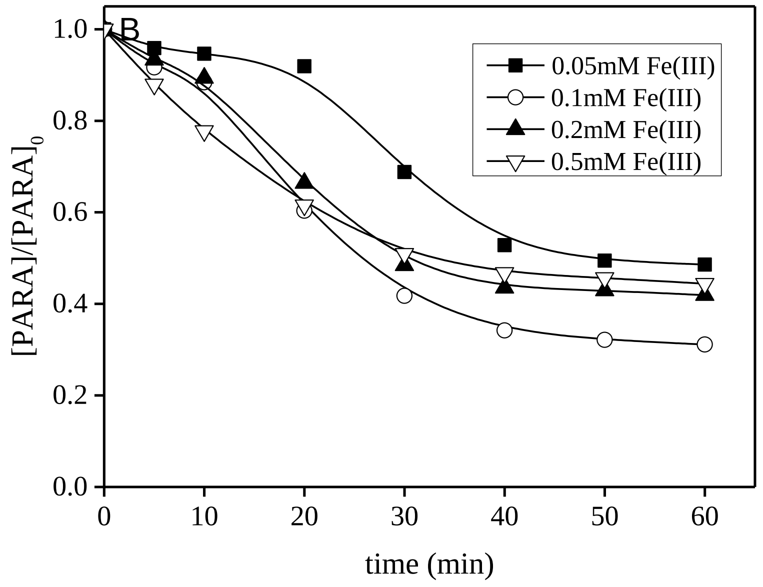

Figure S3. Effect of Fe(III) concentration on PARA degradation with UV irradiation in aerated solutions. Conditions: $[PARA]_0$ = 10 µM, $[S(IV)]_0$ = 1.0 mM, pH_{init} = 7.0.
